# Industrial Efficiency and Fatigue: Overwork and Overstrain

**Published:** 1917-06-16

**Authors:** 


### INDUSTRIAL EFFICIENCY AND FATIGUE.

### Overwork and Overstrain.

The Committee appointed by the Minister of Munitions to inquire into the health of munition workers, having in view the immediate urgency of many of the problems which they were called upon to investigate, have published a series of memoranda. These have been followed by an interim report dealing with industrial efficiency and fatigue, which contains the results of some inquiries concerning output, lost time, sickness, etc., and with which is incorporated the Committee's Memoranda No. 7 ("Industrial Fatigue and its Causes") and No. 12 ("Output in Relation to Hours of Work").

A good deal of attention has been devoted to the hygienic needs of the worker, and to his environment. Little attention has hitherto been paid to industrial fatigue or "staleness," their relationship to output, or their dependence upon hours of work and method of payment. The Committee have taken the evidence of various witnesses whose experience and knowledge should fit them to speak with authority concerning industrial fatigue and others matters affecting the health and efficiency of workers. "These, with hardly an exception, have been unable to point to any exact data in support or in disproof of their particular views." There has been in the past, the Committee consider, a complete absence of systematic effort to collect exact data which might prove of permanent value in the solution of industrial problems.

Fatigue, revealing itself by a diminished capacity for doing work, is not merely the expression of muscular weariness, although the symptoms are often referred to the muscles. Fatigue is a "nervous" phenomenon, the initiating and distributing nervous mechanisms becoming exhausted before the muscles; further, bodily sensations are fallacious as guides to the state of fatigue, and give an inadequate measure of it. Fatigue is measurable at any stage by a diminished capacity for performing the act which caused it, and before its signs appear plainly, or at all, in sensation. In nervous fatigue, then, it may happen that, without obvious sign and without his knowing it himself, a man's capacity for work may diminish owing to unrecognised commencing "nervous" exhaustion. The results, too, of fatigue which advances beyond physiological limits ("over-strain") not only reduce capacity at the moment, but do damage of a more permanent kind, which will affect capacity for periods far beyond the next normal period of rest.

Very interesting questions arise for solution. What is the effect of monotony in work as regards the onset of fatigue? Was the skilled craftsman of the past subject to fatigue, although (carrying his work, as he did, through one process and another to completion) he gained "interest," and was relieved from monotony? Have trade unions' restrictions been blessings in disguise? Has the "slacker" been unconsciously sharing in "a tradition of slowed labour which has necessarily arisen, probably in large part automatically, as a kind of physiological self-protection" during excessively long hours of work? An important and early sign of fatigue in the nervous centres is a want of co-ordination and failure in the power of concentration. It would be supposed that practice and training should result in increased output of work with less effort. This is not always the case.

New hands are now, after six months, producing, in spite of their inexperience, not only 5,000, as expected, but 13,000 of these articles per week. The older hands do not approach this output, though all the mechanical conditions of work are practically equal. As patriotic interest in their output appears to be shared here by all the men alike, the lower output by the more experienced hands appears to be assignable only to the effects of long-standing customary restrictions upon habits or rhythm of work from which the newer hands are free.

The accumulated results of fatigue are damaging to general health, and result in "staleness." This "staleness," which results from "all work and no play," essentially differs from that associated with athletic training. The latter has almost invariably followed upon disturbance of digestive and metabolic processes, due to the attempt to load the system with an excess of highly "nutritious" food, and to reduce the quantity of fluid in the diet. Industrial "staleness," on the other hand, "is attributed almost wholly to persistent long hours and the deprivation of weekly rest. It has grave accompaniments, which paradoxically appear not only in a state of lethargy and indifference, but also in a craving for change and excitement." The condition is one to which brain-workers are certainly no less liable than those whose work is manual and monotonous! A desire for change and excitement exists before the stage of "staleness" is reached, and (as the Committee remark) "hours of labour which give little chance of spending the wages earned diminish the incentive to earn more money."

It is impossible for us in the space at our disposal to carry further the consideration of the Report, which affords much interesting material for study both in the industrial and statistical and the medical sections. In conclusion, the following passage, which bears upon one of the prominent questions of the day, may be quoted:—

This class of work causes the men to become very thirsty . . . the effort called for is tremendous, and the way these men perspire . . . is astonishing. Beer is the usual refreshment. A few of the workers are abstainers from alcohol in any form. These latter are usually the most reliable men. When the supply of drink was restricted by the closing of the public-houses in the district, a great improvement in the health and the time-keeping of the workmen was noticed, and was admitted by the men. No satisfactory substitute for beer, so far as is at present known, has been introduced. The use of such substitutes as oatmeal-water and barley-water is stated to cause skin eruptions and boils.

